# Metformin carbon nanodots promote odontoblastic differentiation of dental pulp stem cells by pathway of autophagy

**DOI:** 10.3389/fbioe.2022.1002291

**Published:** 2022-09-09

**Authors:** Jinjin Lu, Rui Li, Shilei Ni, Yuandong Xie, Xinchen Liu, Kai Zhang, Yi Li

**Affiliations:** ^1^ Department of Pediatric Dentistry, Hospital of Stomatology, Jilin University, Changchun, China; ^2^ Jilin Provincial Key Laboratory of Tooth Development and Bone Remodeling, Jilin University, Changchun, China; ^3^ State Key Laboratory of Supramolecular Structure and Materials, College of Chemistry, Jilin University, Changchun, China

**Keywords:** autophagy, cell differentiation, carbon dots, nanomaterials, odontogenesis

## Abstract

Human dental pulp stem cells (hDPSCs) have been a focus of pulp regeneration research because of their excellent odontogenic potential and availability. Applying the odontoblastic differentiation of hDPSCs to tooth regeneration has been challenging. Metformin-based carbon nanodots (MCDs) were synthesized and characterized to investigate their effects *in vitro* on odontoblastic hDPSC differentiation and the underlying mechanism. MCDs were synthesized by a hydrothermal treatment method and characterized using transmission electron microscopy (TEM), Fourier transform infrared spectroscopy, and X-ray photoelectron spectroscopy. The biocompatibility and fluorescence properties of the MCDs in Dulbecco’s modified Eagle’s medium high-glucose culture medium and the *in vitro* odontogenic potential and related mechanism of the bioactive nanomaterial was explored. TEM images showed that MCDs were spherical in shape with a size of approximately 5.9 nm. MCDs showed biological safety in cell viability, apoptosis, and fluorescence labelling ability at a concentration up to 200 μg/ml *in vitro*. The presence of MCDs facilitated high-efficiency odontogenic differentiation of hDPSCs by promoting odontogenic gene and protein expression. Moreover, MCDs promoted odontoblastic hDPSC differentiation *via* autophagy. MCDs are capable of activating autophagy and enhancing the odontogenic differentiation of hDPSCs by upregulating odontoblast gene marker (DMP1, DSPP, RUNX2, and SP7) and protein (DSPP and DMP1) expression.

## Introduction

Dental pulp regeneration is an ideal solution for destructive diseases of the dental pulp, such as pulpitis and pulp necrosis. Human dental pulp stem cells (hDPSCs) are prime candidates for such treatment, given their multipotent differentiation potential and lineage specification into odontoblasts (Yang et al., 2012; Mozaffari et al., 2019). To achieve functional pulp regeneration, hDPSCs need to be differentiated into different types of cells, such as odontoblasts, fibroblasts, and vascular endothelial cells (Sui et al., 2019; Ahmed et al., 2020). Studies have focused on exploring new biomaterials to manipulate hDPSC differentiation, particularly in promoting odontogenic differentiation, which is crucial for dentin regeneration, as dentin is a specific hard tissue of great significance in increasing the mechanical resistance of the root and building the dental pulp defensive system (Ahmed et al., 2020). However, applying bioactive materials to orchestrate hDPSC differentiation into odontoblasts, which specifically generate dentin, remains a challenge.

Recently, carbon dots (CDs), defined as nanoparticles <10 nm in size, which are easily surface-functionalized, have demonstrated the ability to promote mesenchymal stem cell differentiation (Shao et al., 2017; Wang et al., 2019a; Han et al., 2019; Meng et al., 2019; Zhang et al., 2020). CDs synthesized from medications hold great promise for biological application, as they not only show excellent biocompatibility, water solubility, and fluorescence but also may maintain and even enhance the original medication effect (Liu et al., 2017; Wang et al., 2019a; Han et al., 2019; Meng et al., 2019). For example, CDs prepared from zinc gluconate, magnesium gluconate, adenosine, and aspirin as raw materials all promote osteogenic differentiation of bone marrow mesenchymal stem cells, in the absence of any external osteo inductive factors (Wang et al., 2019b; Han et al., 2019; Meng et al., 2019). However, limited data are available on the effect of such CDs on hDPSCs.

**FIGURE 1:**
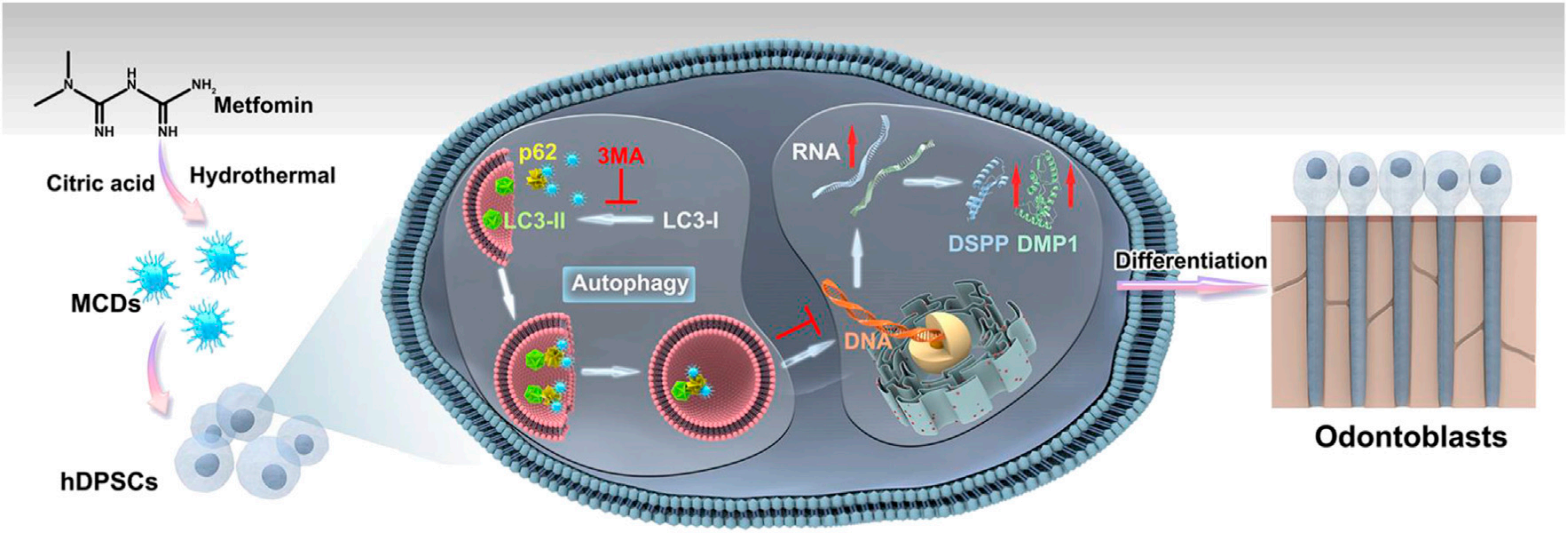
Experimental design—analytical workflow. Metformin-based carbon nanodots (MCDs) were synthesized by a hydrothermal treatment method and characterized using transmission electron microscopy (TEM), Fourier transform infrared spectroscopy, and X-ray photoelectron spectroscopy. Moreover, MCDs are capable of activating autophagy and enhancing the odontogenic differentiation of human dental pulp stem cells (hDPSCs) by upregulating odontoblast gene marker (DMP1, DSPP, RUNX2, and SP7) and protein (DSPP and DMP1) expression.

As a first-line medication for type 2 diabetes, metformin exhibits pleiotropic effects on a variety of tissues (Lv and Guo, 2020). Qin’s team has reported metformin’s potential odontogenic effect by enhancing hDPSCs differentiation *via* the AMPK signal pathway, and designed novel calcium phosphate cement with metformin-loaded chitosan for odontogenic differentiation of hDPSCs (Qin et al., 2018a; Qin et al., 2018b). Resultantly, we propose that metformin-based CDs (MCDs) could be effective and safe odontoinductive agents for deriving odontoblasts from hDPSCs. Herein, we aimed to first synthesize MCDs and then employ them to investigate the effects of MCDs on the biocompatibility and odontoblastic differentiation of hDPSCs *in vitro* (Figure 1).

## Materials and methods

### Materials

Metformin and citric acid were purchased from Shanghai Aladdin Chemicals (Shanghai, China). KCl, HCl, NaOH, Na_2_SO_4_, MgSO_4_, CaSO_4_, Fe_2_(SO_4_)_3_, CuSO_4_, ZnSO_4_, and AlCl_3_ were purchased from Beijing Chemical Reagent Co. (Beijing, China). Dulbecco’s modified Eagle’s medium high glucose (H-DMEM) and antibiotics (100 U/ml penicillin and 100 U/ml streptomycin) were purchased from Hyclone (Logan, UT, United States). Fetal bovine serum (FBS), phosphate-buffered saline (PBS), and TRIzol reagent were purchased from (Gibco, Waltham, MA, United States). Crystal violet staining reagent was purchased from Solarbio (Beijing, China). The 3-(4,5-dimethylthiazol-2-yl)-2,5-diphenyltetrazolium bromide (MTT) was purchased from Sigma‒Aldrich (St Louis, MO, United States). An Annexin V APC apoptosis analysis kit was purchased from Sungene Biotech (Tianjin, China). All antibodies, except DMP1 and DSPP, were purchased from Proteintech Group Laboratory (Wuhan, China). DMP1 and DSPP antibodies were purchased from ImmunoWay Biotechnology Co. (Plano, TX, United States) and Santa Cruz Biotechnology (Dallas, TX, United States), respectively. CD44-PE, CD90-PE, CD45-FITC, and CD11b-FITC antibodies were purchased from BioLegend (San Diego, CA, United States). 3-Methyladenine (3-MA) was purchased from Topscience (Shanghai, China). Enhanced chemiluminescence, the Hieff First-Strand cDNA Synthesis Super Mix for the RT-qPCR kit, and the Hieff qPCR SYBR Green Master Mix kit were purchased from Yeasen Biotech (Shanghai, China).

### Synthesis of MCDs

MCDs were synthesized by a one-pot hydrothermal process, as previously described (Liu et al., 2017). Briefly, 1 mmol each of metformin and CA were dissolved in 10 ml of deionized water. The mixture was then transferred to a poly (tetrafluoroethylene) (Teflon)-lined autoclave (25 ml) and heated to 200°C for 4 h. The product was cooled naturally to room temperature. Large impurities in the product were removed with a 0.22 μm filter, and the dark brown solution obtained was transferred to a 500–1,000 Da dialysis bag against deionized water for 24 h to remove excess precursors and by-products. MCD powder was collected after freeze-drying.

### Characterization of MCDs

Transmission electron microscopy (TEM) was performed using a JEM-2100F microscope (JEOL, Tokyo, Japan). Photoluminescence (PL) spectra were recorded on an RF-5301 PC spectrophotometer (Shimadzu Corp, Kyoto, Japan). Ultraviolet-visual light (UV-vis) absorption spectra were measured on a Shimadzu 3100 UV-Vis spectrophotometer. Fourier transform infrared (FT-IR) spectra were recorded on a Nicolet AVATAR 360 FTIR spectrophotometer (Thermo Fisher Scientific; Waltham, MA, United States). X-ray photoelectron spectroscopy (XPS) analysis was performed on an ESCALAB 250 spectrometer (Thermo Fisher Scientific) with a monochromatic Al Kα excitation source (1,486.6 eV).

### Isolation, cultivation, and characterization of hDPSCs

hDPSCs were isolated, cultured, and characterized as previously described (Gronthos et al., 2002). The procedure was approved by the Ethics Committee of the Hospital of Stomatology, Jilin University. Cells were isolated from the third molar of healthy individuals (18–25 years old) undergoing tooth extraction for orthodontic treatment. The isolated cells were maintained in H-DMEM containing 20% FBS and antibiotics, at 37°C in a humidified 5% CO_2_ atmosphere. When the primary cell culture reached 80% conﬂuence, the cells were trypsinized and subcultured in H-DMEM with antibiotics and 10% FBS. The cells from the second passage were washed and resuspended in PBS and incubated with anti-human antibodies, including CD44-PE, CD90-FITC, CD45-PE, and CD11b-FITC antibodies, for 30 min at 4°C, in the dark, to examine their surface antigen expression. The cell samples were then evaluated by flow cytometry (FACS calibur; Becton Dickinson Biosciences, Franklin Lakes, CA, United States). hDPSCs from the third to fifth passages were used for subsequent experiments. Supplementary Figures S1A,B present microscope images of hDPSCs after 10 days of primary passage and in the third passage, respectively. Consistent with other mesenchymal stem cell populations, hDPSCs expressed high levels of mesenchymal markers and low levels of hematopoietic markers (Supplementary Figure S1C) (Cui et al., 2018).

### Cytotoxicity assessment and apoptosis assay

MTT assays were used to detect the cytotoxic effects of MCDs. First, 200-μl aliquots with a density of 2×10^3^ cells/mL were placed in each well of a 96-well plate and were then treated with MCD concentrations of 50, 100, 200, 400, and 800 μg/ml, followed by incubation for 1, 3, 7, 10, and 14 days at 37°C in a humidified 5% CO_2_ atmosphere. The medium was replaced with 180 μl PBS and 20 μl of stock MTT (5 mg/ml), after which the cells were incubated for another 4 h. Then, the medium was removed, and the dark blue formazan crystals that had formed were dissolved in DMSO. Absorbance was measured at 490 nm using a microplate reader in the shaking mode (RT-6000; Lei Du Life Science and Technology Co., Shenzhen, China). Untreated cells were used as a control to determine the relative cell viability.

Apoptosis analysis was performed using an Annexin V APC apoptosis analysis kit. Briefly, hDPSCs were seeded onto 6-well plates at a seeding density of 3×10^5^ cells/well. Cells (1×10^5^) from the 50, 100, 200, 400, and 800 μg/ml MCD-treated groups were collected at 7 days, washed with PBS, and resuspended in 100 μl of binding buffer. Annexin V APC (5 μl) and propidium iodide solution (5 μl) were added to the cell solution for 15 min at room temperature, followed by flow cytometry analysis within 1 h.

### Cell labelling

hDPSCs were seeded onto clean coverslips in a 6-well plate at a density of 1×10^5^ cells/well for 24 h. Subsequently, the cells were treated with 50, 100, 200, 400, and 800 μg/ml MCD in medium for another 24 h and washed three times with PBS. Finally, the cell imaging under ultraviolet excitation wavelengths were observed under an inverted fluorescence microscope (Olympus, Tokyo, Japan). Supplementary Figures S2 shows the bright blue colour within the cytoplasm when hDPSCs were incubated with MCDs, indicating that MCDs might be applied to track the dynamic changes of cells through fluorescence.

### Real-time polymerase chain reaction

The cell samples were collected and washed twice with PBS on ice. Total RNA was extracted using TRIzol reagent, according to the manufacturer’s protocol. DNase I-treated RNA (1 µg) was used for the reverse transcription reaction with a Hieff First Strand cDNA Synthesis Super Mix for RT-qPCR. Quantitative PCR analysis was performed using the Hieff qPCR SYBR Green Master Mix kit. The expression of each investigated gene was normalized to that of the housekeeping gene β-actin, and fold-differences were calculated using the comparative Ct method. The odontogenic markers were *DSPP, DMP1, RUNX2, SP7,* and *COL1*. The following primer sequences, generated by Sangon Biotech (Shanghai, China), were used: *β-actin* (forward primer: 5′-TGG​CAC​CCA​GCA​CAA​TGA​A-3′, reverse primer: 5′-CTA​AGT​CAT​AGT​CCG​CCT​AGA​AGC​A-3′); *DSPP* (forward primer: 5′-GCT​GGA​AGC​AAT​AAC​AGT​ACA​G-3′, reverse primer: 5′-TGC​TGT​TGA​TCT​GAG​GTG​TTA​T-3′); *DMP1* (forward primer: 5′-CAA​AGA​AGA​TAG​CAA​CTC​CAC​G-3′, reverse primer: 5′-CAT​CAA​CTG​TTA​ATT​TCC​GGC​T-3′); *RUNX2* (forward primer: 5′-CAC​TGG​CGC​TGC​AAC​AAG​A-3′, reverse primer: 5′-CAT​TCC​GGA​GTC​AGC​AGA​ATA​A-3′); *SP7* (forward primer: 5′-TGG​CGT​CCT​CCC​TGC​TTG-3′, reverse primer: 5-'TGC​TTT​GCC​CAG​AGT​TGT​TG-3′); and *COL1* (forward primer: 5′-TGT​TGG​TCC​TGC​TGG​CAA​GAA​TG-3′, reverse primer: 5′-GTC​ACC​TTG​TTC​GCC​TGT​CTC​AC-3′). Data were calculated using the 2^−ΔΔCT^ method.

### Western blotting

The cell samples were harvested, washed with PBS on ice, and subsequently lysed in lysis buffer containing a protease inhibitor cocktail. Proteins were separated on sodium dodecyl sulfate-polyacrylamide gels and transferred to polyvinylidene fluoride membranes. The membranes were incubated overnight with primary antibodies against LC3 at 1:500, p62 at 1:1,000, Beclin1 at 1:1,000, DMP1 at 1:500, DSPP at 1:1,000, and GAPDH at 1:5,000 dilution. Subsequently, the membranes were incubated with secondary antibodies for 60 min, and blotted bands were visualized using enhanced chemiluminescence.

### Transmission electron microscopy

hDPSCs were cultured with 50 μg/ml MCD for 24, 48, or 72 h, fixed with 2% glutaraldehyde for 1 h at 4°C, then incubated in PBS overnight, and post-fixed in 1% osmium tetroxide in the same buffer for 2 h at room temperature. After dehydration with 50, 70, 90, and 100% ethanol, the samples were embedded in Epon. Ultrathin sections of cells were cut using a Leica EMUC6 ultramicrotome (Leica Biosystems, Wetzlar, Germany) and sections were settled on 200-mesh carbon-coated copper grids. After staining with 2% uranyl acetate and lead citrate, the samples were observed using an EM-14100BINC TEM (JEOL, Tokyo, Japan) at 80 kV. TEM images were taken to observe autophagic vesicles.

### Autophagy inhibitor

To evaluate whether MCDs promote odontogenic differentiation of hDPSCs *via* autophagy, autophagy was blocked in hDPSCs by adding 3-MA. In this experiment, hDPSCs were treated with 50 μg/ml MCDs for 0, 6, 12, 24, 48, and 72 h. Additionally, 3-MA (0.5 mM) or 25 μg/ml metformin (as control), equivalent to the amount of metformin used for synthesis of 50 μg/ml MCDs, was added to the hDPSCs for 12 h prior to the addition of MCDs. Protein levels of LC3 and p62 were detected by western blotting, with GAPDH protein as a loading control.

### Statistical analysis

Data were analysed using one-way analysis of variance with post hoc Tukey’s test using GraphPad Prism software (GraphPad Software, Inc., San Diego, CA, United States). The level of statistical significance for all tests was set at *p* < 0.05.

## Results

### Characterization of CDs

**FIGURE 2:**
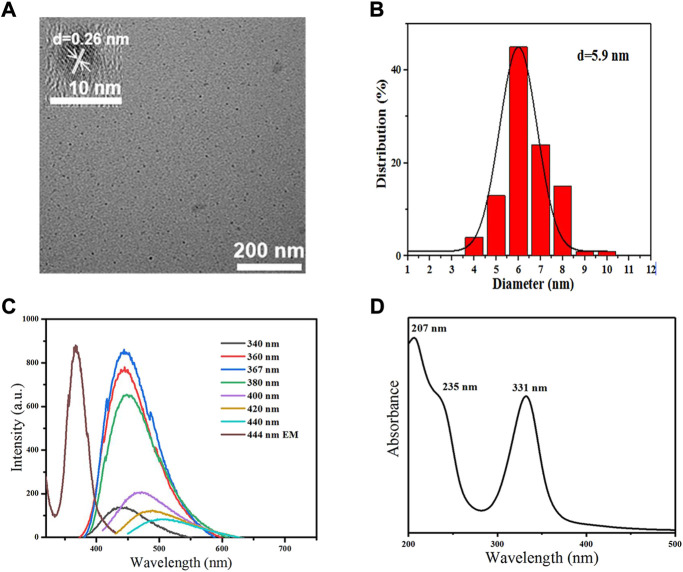
Characterization of MCDs. **(A)** TEM images of MCDs (scale bar: 200 nm); the inset is the HRTEM image of MCDs (scale bar: 10 nm). **(B)** Size distribution histogram of MCDs. **(C)** PL spectra of MCDs with different excitation wavelength and emission spectra of MCDs **(D)** UV-vis absorption of MCDs. HRTEM: high-resolution transmission electron microscopy, MCDs: metformin-based carbon nanodots, PL: photoluminescence, TEM: transmission electron microscopy, UV-vis: ultraviolet-visible.

TEM images illustrated that MCDs had good dispersibility and exhibited a spherical particle shape. High-resolution TEM of MCDs showed a distinct lattice structure with a lattice spacing of 0.26 nm (Figure 2A). The average particle size was approximately 5.9 nm (Figure 2B). The PL spectrum (Figure 2C) and the interpolated images revealed that an aqueous solution of the CDs exhibited strong blue fluorescence with an emission peak at 444 nm and an optimum excitation wavelength of 367 nm. The UV-vis absorption spectrum (Figure 2D) of the MCDs contained a distinct absorption peak at 332 nm corresponding to the n→π* transition of the C=O/C=N chemical bond (Feng et al., 2017). The peak at 232 nm contributed to the π→π* transition of the aromatic C=C bond (Yang et al., 2014; Liu et al., 2017).

**FIGURE 3:**
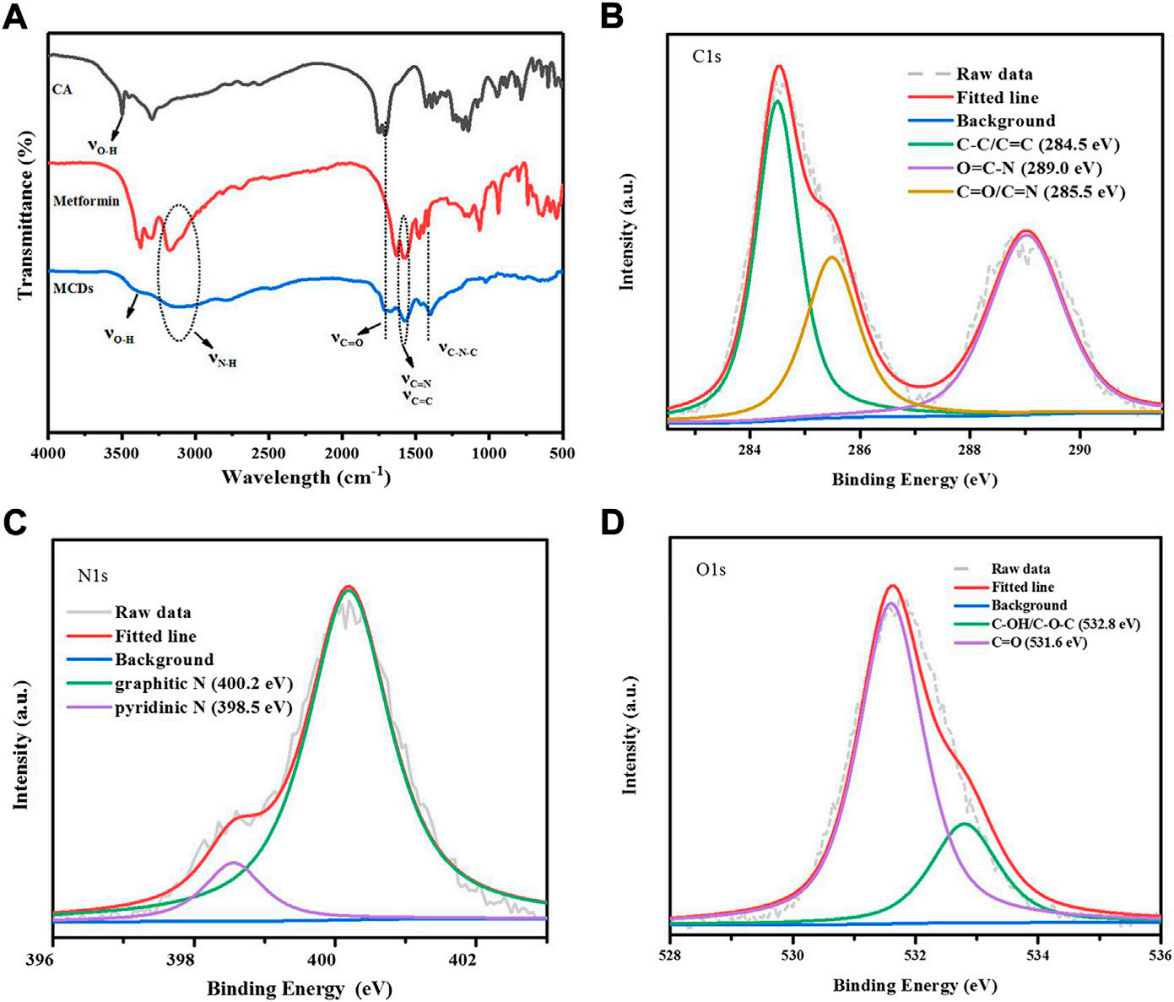
**(A)** FT-IR spectra of CA, MH, and MCDs, respectively. XPS analysis of MCDs. High resolution C1s **(B)**, N1s **(C)**, and O1s **(D)** peaks of MCDs. FT-IR: Fourier transform infrared, MCDs: metformin-based carbon nanodots, XPS: X-ray photoelectron spectroscopy.

FT-IR spectroscopy (Figure 3A) was performed to characterize the chemical groups of MCDs, and the following peaks were observed: stretching vibration of O–H at 3,300 cm^−1^, N–H and 3,115 cm^−1^, C=O at 1,680 cm^−1^, C=C/C=N at 1,500–1,620 cm^−1^, and C–N–C at 1,400 cm^−1^ (Feng et al., 2017; Liu et al., 2018; Yan et al., 2019), which revealed that the MCDs retained the chemical groups of metformin.

XPS (Supplementary Figure S3A) was employed to further investigate the chemical structures of the MCDs. The C1s spectra (Figure 3B) showed three peaks of C–C/C=C (284.5 eV), O=C–N (289.0 eV), and C=O/C=N (285.5 eV). The N1s spectra (Figure 3C) showed two peaks: graphitic N (398.5 eV) and pyridinic N (400.2 eV). The O1s spectra (Figure 3D) appeared as two peaks of C–OH/C–O–C at 532.8 eV and C=O at 531.6 eV (Narayanan et al., 2006; D'Souza et al., 1997; Bae et al., 2018; Li et al., 2011), consistent with the FT-IR results. The above data indicated that the MCDs were rich in functional groups.

The photostability of the MCDs was also evaluated before the biological experiments. MCDs showed excellent fluorescence stability in different metal ion solutions (Supplementary Figure S3B). The PL intensity remained nearly unchanged in the pH range of 5–14 (Supplementary Figure S3C) and in high-concentration KCl solution (Supplementary Figure S3D), indicating that MCDs were applicable in the bio-microenvironment.

### Biocompatibility of MCDs

**FIGURE 4:**
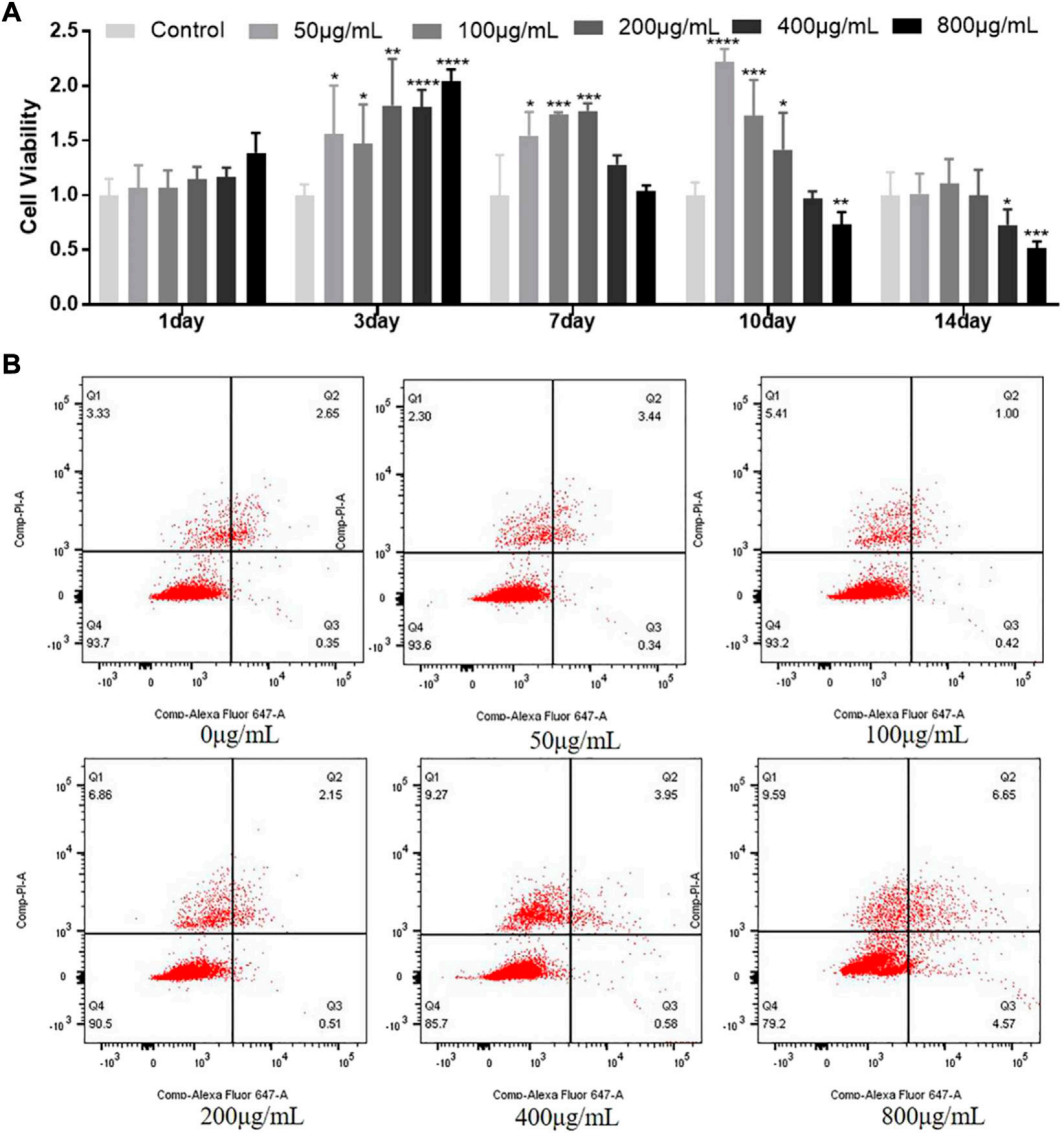
Biocompatibility of MCDs. **(A)** The relative viability of hDPSCs after incubation with MCDs at various concentrations for 1, 3, 7, 10, and 14 days. **(B)** Apoptotic ratio of hDPSCs after incubation with MCDs at various concentrations for 7 days were assessed by Annexin V-APC assay and measured by fluorescence-activated cell sorting analysis. hDPSCs: human dental pulp stem cells, MCDs: metformin-based carbon nanodots. MCDs promote the odontogenic differentiation of hDPSCs.

As shown in Figure 4A, MCDs at concentrations of 50, 100, and 200 μg/ml significantly promoted cell proliferation at 3, 7, and 10 days, while concentrations of 400 and 800 μg/ml MCDs inhibited cell proliferation at 10 and 14 days. Moreover, we determined whether the same concentration could induce apoptotic cell death using Annexin VAPC assessment by flow cytometry (Figure 4B). The group of MCDs concentrations up to 400 μg/ml showed no significant increase in apoptosis observed at 7 days, as compared with the control group. However, MCDs at concentrations of 800 μg/ml showed an increase in early apoptosis. Cumulatively, these results demonstrated that the effects of MCDs on hDPSC viability and apoptosis were dose-dependent. MCD concentrations of 50, 100, and 200 μg/ml exhibited minimal cytotoxicity in hDPSCs.

### MCDs promote the odontogenic differentiation of hDPSCs

**FIGURE 5:**
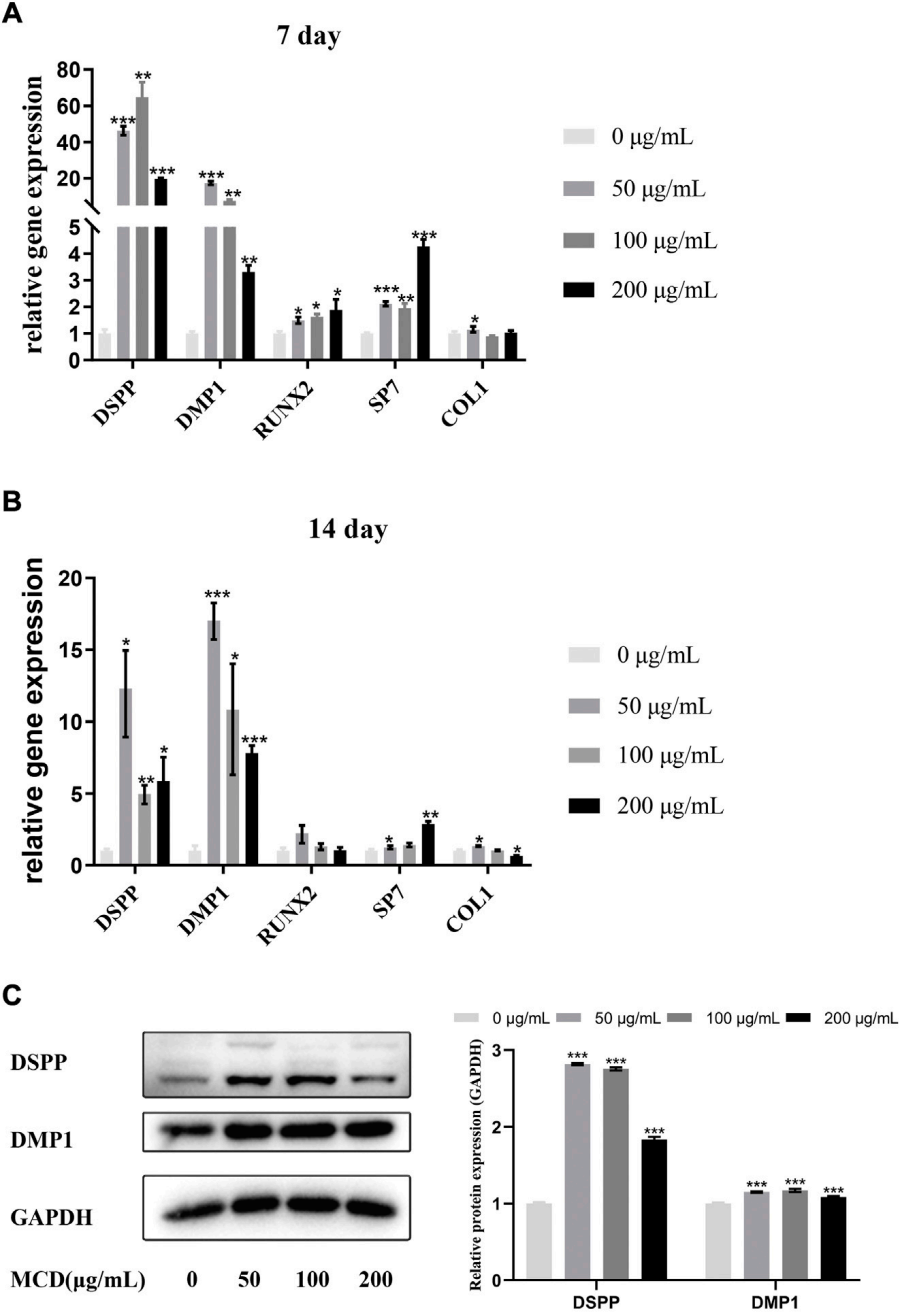
The effects of MCD treatment on the odontoblastic differentiation of hDPSCs for 7 and 14 days. **(A,B)** The messenger RNA expressions of DSPP, DMP1, Runx2, SP7, and Col1 were analyzed using real-time reverse transcription polymerase chain reaction. These data represent three independent experiments and are expressed as the mean ± SD. (*) *p* < 0.05, (**) *p* < 0.01, and (***) *p* < 0.001 vs. control. **(C)** DMP1 protein and DSPP protein expression after 14 days exposure to MCDs. GAPDH protein expression served as a loading control. hDPSCs: human dental pulp stem cells, MCD: metformin-based carbon nanodot.

The gene expression levels of odontoblastic markers, such as dentin matrix protein 1 (*DMP1*), dentin sialo phosphoprotein (*DSPP*), runt-related transcription factor 2 (*RUNX2*), specificity protein 7 (*SP7*), and type 1 collagen (*COL1*) were measured to investigate the effects of MCDs on the odontoblastic differentiation of hDPSCs at 7 and 14 days (Figures 5A,B). The expression levels of *DSPP*, *DMP1*, and *SP7* were significantly increased (*p* < 0.05) in all MCD-treated groups at 7 and 14 days, indicating that MCDs promoted the differentiation of hDPSCs into odontoblasts. The expression of *RUNX2* increased significantly (*p* < 0.05) at 7 days and did not increase further after 14 days. The results clearly demonstrated that MCDs significantly enhanced the expression of odontogenic genes, thus preliminarily proving that MCDs can promote the odontogenic differentiation of hDPSCs.

To determine the effects of MCDs on odontogenic differentiation further, we detected specific odontogenic proteins, including *DSPP* and *DMP1*, at 14 days (Figure 5C). The expression of DSPP and *DMP1* in the MCD-treated group was significantly higher than that in the control group, which was coordinated with the odontogenic marker gene expression at day 14. Notably, 50 μg/ml MCDs were the most effective in promoting *DSPP* and *DMP1* expression, while the expression in the 200 μg/ml MCD group was significantly lower than that in the other MCD groups. These results reinforce the hypothesis that MCDs promote odontogenic differentiation by upregulating the expression of odontogenic-specific markers. Given the odontogenic gene expression pattern at day 14, a lower concentration of MCDs at 50 μg/ml, was the most effective in promoting odontogenic differentiation.

### MCDs promote the odontogenic differentiation of hDPSCs through autophagy

**FIGURE 6:**
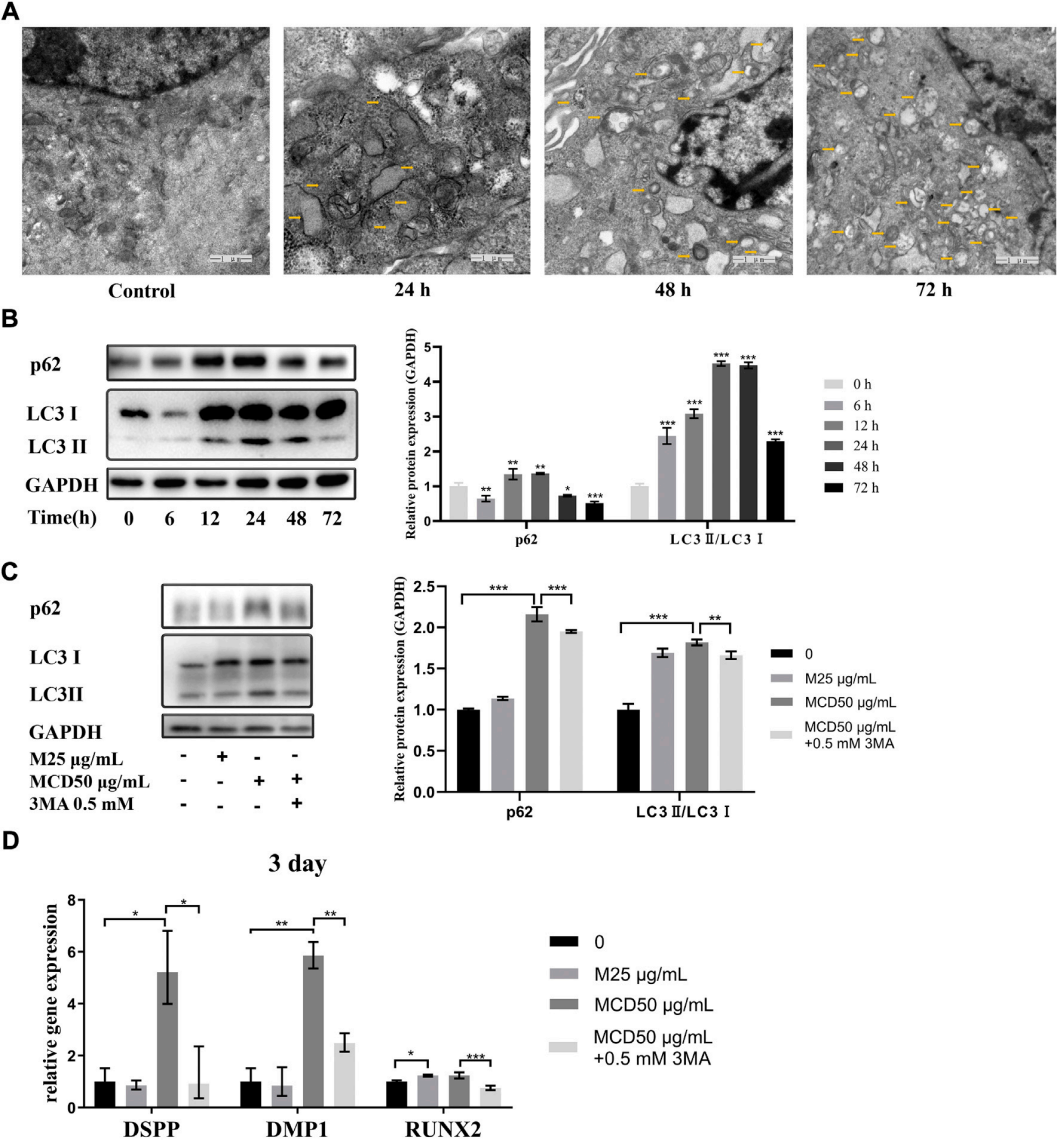
MCDs promote odontogenic differentiation of hDPSCs *via* autophagy. **(A)** TEM images of autophagic vesicles (yellow arrows) in 0, 24, 48, and 72 h (scale bar: 1 μm). **(B)** The hDPSCs were treated with 50 μg/ml MCDs for 0, 6, 12, 24, 48, and 72 h, and protein levels of LC3 and p62 were detected. **(C)** hDPSCs were treated with 3-MA (0.5 mM) for 12 h prior to addition of 50 μg/ml MCDs for 3 days. The protein levels of LC3 and p62 were detected. GAPDH protein expression served as a loading control. **(D)** hDPSCs were treated with 3-MA (0.5 mM) for 12 h prior to addition of 50 μg/ml MCDs for 3 days. The messenger RNA expression of DSPP, DMP1, Runx2 was analyzed using real-time reverse transcription polymerase chain reaction. These data represent three independent experiments and are expressed as the mean ± SD. (*) *p* < 0.05, (**) *p* < 0.01, and (***) *p* < 0.001 vs. control. hDPSCs: human dental pulp stem cells, MCDs: metformin-based carbon nanodots, 3-MA: 3-Methyladenine.

To determine the mechanism by which MCDs promote odontogenic differentiation, we used TEM and western blotting to detect the autophagy-related structures and protein expression of hDPSCs. Compared to that in the controls, TEM showed typical autophagosomes in hDPSCs when the cells were cocultured with 50 μg/ml MCDs for 24 h, which increased consistently until 72 h (Figure 6A). The western blotting results showed that the expression of p62 and LC3Ⅱ was significantly increased at 12 h and peaked at 24 h (Figure 6B). Subsequently, the expression of p62 decreased. Combined with the formation of autophagosomes detected by TEM, the results indicated that MCDs were effective bioactive nanoparticles for activating autophagy in hDPSCs.

We investigated the relationship between MCD-induced autophagy and odontoblastic differentiation. As shown in Figures 6C,D, 50 μg/ml MCDs successfully increased the expression of p62 and LC3Ⅱ and promoted the expression of odontoblastic differentiation markers. When the hDPSCs were pretreated with 3-MA for 12 h, accompanied by a significant decrease in odontogenic gene expression, the expression of p62 and LC3Ⅱ also decreased after 72 h, by which time there was no difference from the control groups. The blockade of MCD-induced autophagy led to a reduction in odontogenic gene expression. The results clearly demonstrated that MCDs promoted odontogenic differentiation of hDPSCs through autophagy. After blocking autophagy, the ability of odontogenic differentiation was inhibited, thus proving that MCDs enhance odontogenic differentiation through autophagy.

## Discussion

Bioactive nanomaterials have been widely proposed for regulating hDPSC differentiation into odontoblasts for pulp regeneration. However, as a novel nanomaterial, CDs were first discovered to promote odontoblastic differentiation *via* autophagy. Through preliminary experiments, we found that CDs could not be synthesized from metformin alone; hence, we introduced citric acid as a regular carbon source. Then, we synthesized MCDs, which had good dispersibility and exhibited a spherical particle shape of 5.9 nm in size, excitation dependence, and rich functional groups. MCDs also showed biological safety in terms of cell viability, apoptosis, and fluorescence labelling ability at a concentration up to 200 μg/ml *in vitro*. Importantly, the presence of MCDs facilitated high-efficiency odontogenic differentiation of hDPSCs by promoting the expression of odontogenic genes and proteins. Notably, we further revealed that MCDs promoted odontoblastic differentiation of hDPSCs *via* autophagy.

The MCDs exhibited typical CD properties, similar to those of other functional CDs, given its rich functional groups. For example, its hydroxyl and amino groups can promote water solubility, and its imine bonds allow easy surface modification. Moreover, the MCDs retained nitrogen-hydrogen bonds in the metformin structure. Regarding its fluorescence properties, we proved its cell labelling, as shown in Supplementary Figure S3.

Good biocompatibility is a prerequisite for the application of nanomaterials in cell function regulation. Therefore, the effect of MCDs on cell viability was assessed using an MTT assay. We found that concentrations of MCD up to 200 μg/ml did not affect cell viability. Moreover, we employed an apoptotic cell death assay to verify this, because Annexin V-APC assessment is more sensitive than MTT assays and can identify apoptosis in the same cell population by flow cytometry. We found that MCDs under 400 μg/ml did not affect apoptosis, and 800 μg/ml showed significantly increased apoptosis. We can conclude that MCDs concentrations under 200 μg/ml exhibited good biocompatibility in hDPSCs.

To investigate the effect of MCD on odontogenic differentiation, we investigated odontogenic biomarkers. DMP1 has been reported to be present during differentiation of hDPSCs and to regulate the expression of DSPP during the early differentiation of odontoblasts (Narayanan et al., 2006). DSPP is also important for specifying odontoblastic differentiation (D'Souza et al., 1997). SP7 (osterix) is required for the proliferation and differentiation of odontoblasts (Bae et al., 2018). RUNX2 is an essential transcription factor that enhances early differentiation of odontoblasts and inhibits odontoblast terminal differentiation (Li et al., 2011; Li et al., 2021; Li et al., 2022), which increases *RUNX2* expression (*p* < 0.05) at 7 days. COL1 is a key protein in dentin formation and repair (Ni et al., 2018). The gene expression levels of *DSPP, DMP1, COL1*, and *SP7* increased significantly at 7 days, indicating that MCDs promoted the differentiation of hDPSCs into odontoblasts. The expression of *RUNX2* increased at 7 days and showed no significant change at 14 days, which may indicate that hDPSCs differentiated into odontoblasts at 14 days. The gene and protein expressions of DSPP and DMP1 increased significantly after 14 days, indicating that the differentiated cells maintained secretory activity and attained dentin function. COL1 expression increased at 7 days but declined at 14 days under MCD 200 μg/ml treatment. We speculated that at day 14, the 200 μg/ml MCDs may have affected cell activity. Thus, we chose the minimum effective concentration of 50 μg/ml to explore the mechanism by which MCD affected cell differentiation.

Autophagy refers to a relatively conservative evolutionary process in which eukaryotic double-membrane vesicles wrap the cell contents and then transport them to lysosomes for degradation (Klionsky et al., 2016). In 2013, Yang et al. (2013) demonstrated the developmental appearance of autophagy during odontogenesis in mouse lower molars, which indicated that autophagy may play an important role in odontogenesis. An increasing number of nanomaterials serve as autophagy modulators (Zhang et al., 2021). The amount of LC3Ⅱ is a common marker for monitoring autophagosome formation, and decreased expression of p62, an autophagy cargo receptor, is often associated with increased autophagic flux (Klionsky et al., 2016). The autophagic flux, which defines the amount of autophagic degradation, indicates autophagic activity. In our study, western blotting results showed that autophagy was activated after the cells were cocultured with MCDs for 12 h, and autophagic flux was enhanced after 24 h. Moreover, blockade of MCD-induced autophagy led to a reduction in odontogenic gene expression. Our findings revealed that MCDs are autophagy activators during the process of odontogenic differentiation. Similar to our research, Schisandrin C, deferoxamine, and midkine have been shown to promote odontogenic differentiation of hDPSCs by enhancing autophagy (Wang et al., 2017; Takanche et al., 2018; Park et al., 2020).

Shao et al. reported that citrate CDs promote osteogenic differentiation of mouse bone marrow mesenchymal cells through the reactive oxygen species (ROS)-mediated MAPK pathway (Shao et al., 2017). ROS can activate autophagy through the MAPK signalling pathway (Wang et al., 2017). Concerning the role of CDs in promoting the differentiation of stem cells, we speculate that CDs may mediate differentiation through similar or even the same signalling pathways, but further studies using citric acid and citrate CDs are required as controls to verify this. Metformin induces autophagy in various cells (Lu et al., 2021). However, in this experiment, metformin alone at 25 μg/ml did not induce autophagy in hDPSCs, and compared with the MCD group, it failed to promote odontogenic marker expression. We hypothesize that the concentration of metformin was too low to exert biological functions in hDPSCs, but this requires further investigation.

## Conclusion

We successfully synthesized bioactive drug CDs, i.e., MCDs. These MCDs could facilitate the odontogenic differentiation of hDPSCs by upregulating the expression of odontoblast gene markers (DMP1, DSPP, RUNX2, and SP7) and proteins (DSPP, DMP1). More importantly, our findings verified that the MCDs exhibited effective odontogenic differentiation through the mechanism of autophagy. To our knowledge, no previous study has shown that CDs are capable of both activating autophagy and enhancing odontogenic differentiation of hDPSCs. Our work highlights the marked potential of these CDs in the thriving fields of regenerative endodontics and autophagy-based cell therapy.

## Data Availability

The original contributions presented in the study are included in the article/Supplementary Material, further inquiries can be directed to the corresponding author.
