# Supplementary material for: Metformin carbon nanodots promote odontoblastic differentiation of dental pulp stem cells by pathway of autophagy
Source: Front Bioeng Biotechnol. 2022 Sep 9;10:1002291. doi: 10.3389/fbioe.2022.1002291 (PMC9506707; doi:10.3389/fbioe.2022.1002291)
Supplement: Supplementary file 1 [file Table1.DOCX]

Supplementary Material

# Supplementary Figures


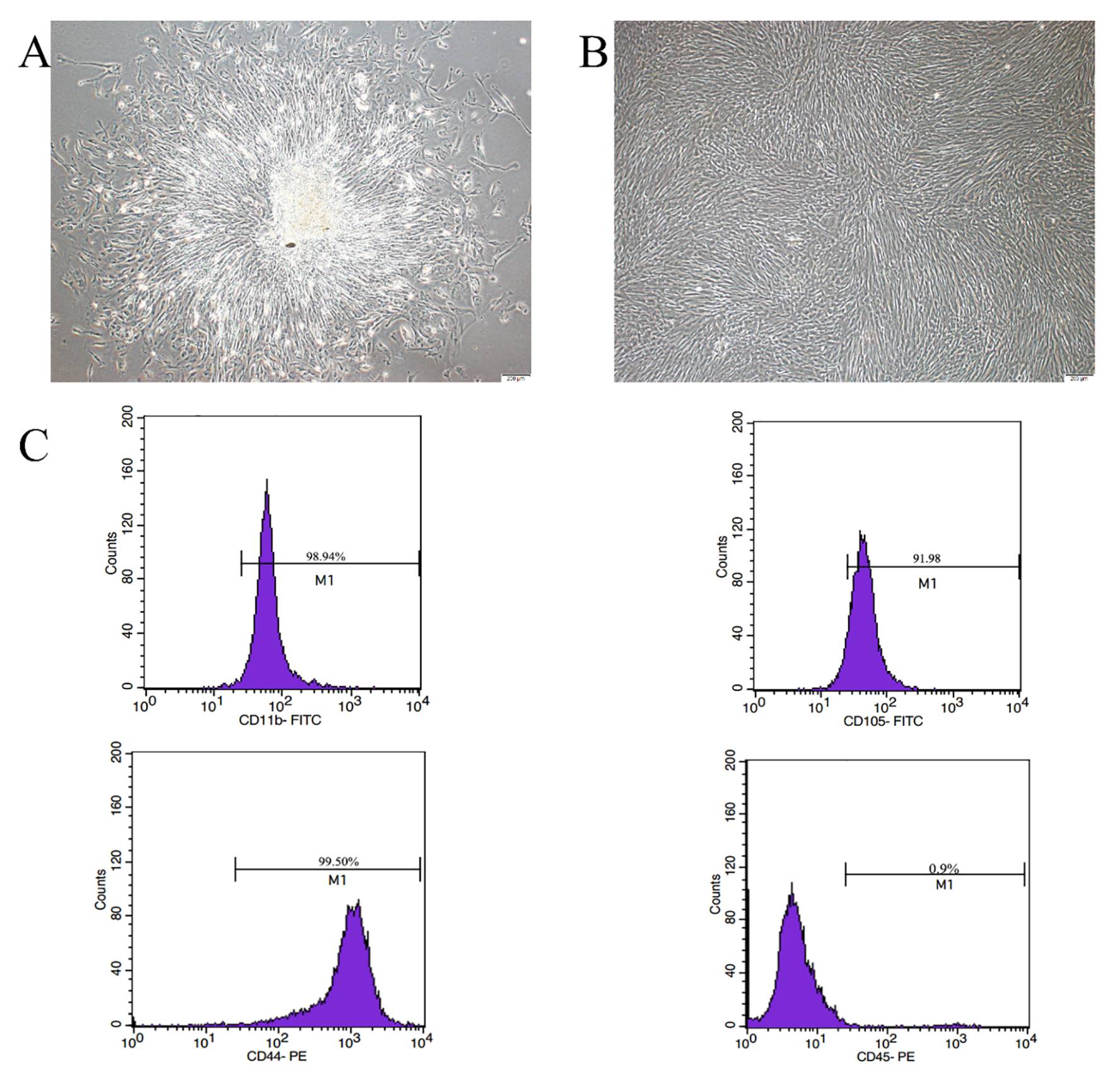


**Supplementary Figure S1.** Culture and identification of hDPSCs. (A) Representative optical microscopic images of hDPSCs at primary passage for 10 days and (B) the third passage (scale bar: 200 μm). (C) hDPSCs phenotype by flow cytometry. The expression of a series of cell surface markers associated with the MSC phenotype was investigated using flow cytometry. Analysis of molecular surface antigen markers in hDPSCs by flow cytometry indicated that cells were negative for CD45, whereas they were positive for CD90, CD11b, and CD44. hDPSCs: human dental pulp stem cells, MSC: metformin-based carbon nanodots.


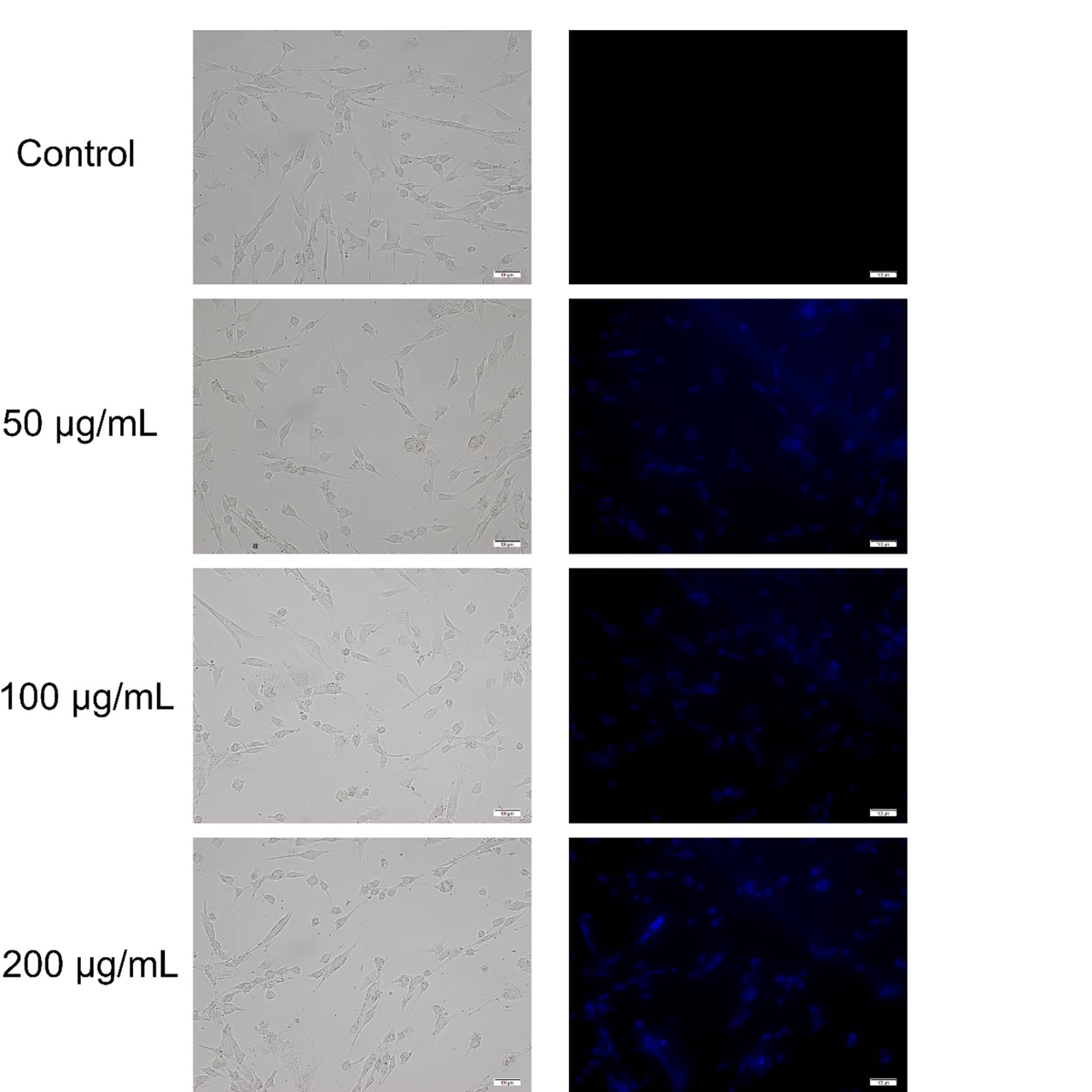


**Supplementary Figure S2.** hDPSCs labeling by MCDs. Images of hDPSCs incubated with 50, 100, and 200 μg/mL of MCDs for 24 h. Blue fluorescence shows location of the probes. Scale bars represent 100 μm. hDPSCs: human dental pulp stem cells, MCDs: metformin-based carbon nanodots.

**
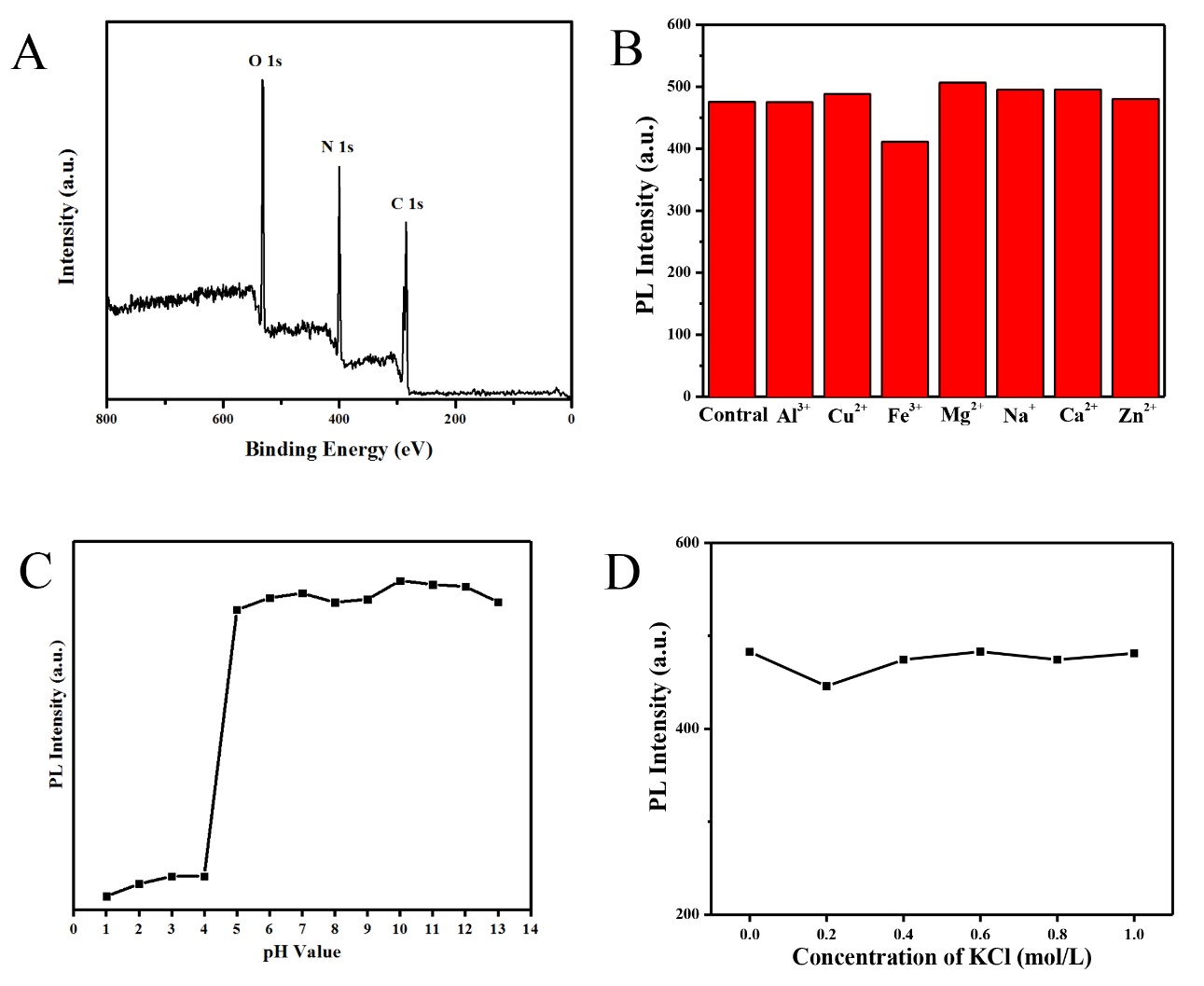
**

**Supplementary Figure S3.** (A) XPS analysis of MCDs in full spectra. (B) Effect of common metal ions on the PL intensity of MCDs. (C) Effect of different solution pH on the PL intensity of MCDs. (D) Effect of different concentration of KCl on the PL intensity of MCDs. MCDs: metformin-based carbon nanodots, PL: photoluminescence, XPS: XPS: X-ray photoelectron spectroscopy.
